# Antibiotic Removal from the Aquatic Environment with Activated Carbon Produced from Pumpkin Seeds

**DOI:** 10.3390/molecules27041380

**Published:** 2022-02-18

**Authors:** İhsan Alacabey

**Affiliations:** Vocational Higher School of Healthcare Studies, Mardin Artuklu University, Mardin 47200, Turkey; ihsanalacabey@hotmail.com or ihsanalacabey@artuklu.edu.tr

**Keywords:** activated carbon, adsorption, ciprofloxacin, pollutant, pumpkin seed, thermodynamics

## Abstract

Antibiotics are among the most critical environmental pollutant drug groups. Adsorption is one of the methods used to eliminate these pollutants. In this study, activated carbon was produced from pumpkin seed shells and subsequently modified with KOH. The adsorbent obtained through this procedure was used to remove ciprofloxacin from aqueous systems. Fourier Transform-Infrared Spectroscopy (FT-IR), Scanning Electron Microscopy (SEM), elemental, X-ray Photoelectron Spectroscopy (XPS), Brunauer–Emmett–Teller (BET) and Zeta analyses were used to characterize the adsorbent. The surface area, in particular, was found to be a very remarkable value of 2730 m^2^/g. The conditions of the adsorption experiments were optimized based on interaction time, adsorbent amount, pH and temperature. Over 99% success was achieved in removal operations carried out under the most optimal conditions, with an absorption capacity of 884.9 mg·g^−1^. In addition, the Langmuir isotherm was determined to be the most suitable model for the adsorption interaction.

## 1. Introduction

Pharmaceutical drugs have been labeled as hazardous class contaminants due to their extensive and long-lived active mechanism on aquatic systems. Antibiotics are a class of drugs used to treat infections caused by sundry microbes, such as certain bacteria and parasites [1,2]. Approximately 30–90% of the antibiotic content introduced into the organism can remain intact and also be actively eliminated from the human or animal bodies [3]. The presence of antibiotics in the environment can trigger antibiotic-resistant bacteria to proliferate even at low concentrations, harming ecological life [4]. Fluoroquinolone (FQ) antibiotics are commonly used in the medical and veterinary fields [5]. Ciprofloxacin (CIP) is one of the most widely administered FQ antibiotics in the world [6]. This antibiotic has been found in concentrations of <1 µg·L^−1^ 1 in streams and wastewater influents and effluents; however, higher concentrations have been found in wastewater from hospitals (3–87 µg·L^−1^) and drug manufacturing facilities (31 mg·L^−1^) [7]. Therefore, the disposal of CIP residues is a critical environmental issue.

Many applications have been used to treat effluents rich in FQs, such as electrochemical oxidation [8], biodegradation [9], photodegradation [10], catalytic degradation [11], micro-extraction [12], oxidation (catalytic degradation) [13] and adsorption [14]. Among current methods, adsorption has proven to be a simple, high-performance and low-cost approach for removing low concentration FQ contaminants from aqueous media [15]. Adsorption is a widely used practice for removing a wide range of FQ contaminants due to its simple planning, practical handling and relatively effortless regeneration. The term “activated carbon” refers to a family of highly porous, amorphous carbon materials [16]. It is a useful adsorbent in removing FQs when compared to zeolite [17], clay [18], silica [19] and carbon nanotube [20] due to its vast surface area microporous structure and superior adsorption capacity [21]. However, due to its high cost, it is not widely used. For this reason, low-cost alternative methods of carbon processing are being investigated, with plant-based wastes coming to the fore in this regard.

Pumpkin is one of the most important vegetables grown around the world. It is a gourd-like squash and belongs to the Cucurbitaceae family. Pumpkin seeds, also known as pepitas, are flattened and vary in size, shape and color. Oil extracted from pumpkin seeds has various benefits such as anti-microbial, anti-fungal and anti-viral properties [22]. The pumpkin seed shell survey shows that many researchers are focusing on the preparation and usage of pumpkin seed shell as a possible absorbent for contaminant removal [23,24,25,26].

The activation of activated carbon using KOH has resulted in the development of ideal materials, particularly for biotechnology. Huang et al. measured the CO_2_ adsorption performance of activated carbons made from a hydrochar taken from garlic peel activated with KOH [27]. Van et al., on the other hand, investigated the efficiency of activated carbon obtained from rice husk in electrochemical studies [28].

The CIP drug was removed from aqueous systems using KOH modified pumpkin seed shells in this investigation. The aim of this study is to demonstrate that activated carbon obtained from pumpkin seed shells can effectively remove drug waste from aqueous systems. In practical applications using the batch system adsorption technique, adsorption conditions were optimized in terms of adsorbent amount, interaction time, pH and temperature.

## 2. Materials and Methods

### 2.1. Materials

Haver Farma Pharmaceutical Joint Stock Company provided the ciprofloxacin used in this study (selflex ciprasel 400 mg/200 mL i.v. infusion solution). Hydrochloric acid (HCl), sodium hydroxide (NaOH) and potassium hydroxide (KOH) were purchased from Sigma-Aldrich (St. Louis, MO, USA). All of the substances used in the experimental studies are of analytical purity.

### 2.2. Preparation of the Adsorbent

To begin, the raw sample was carbonized for one hour at a rate of 10 °C/min at 500 °C. The charred sample was combined with (1:4) KOH and a sufficient amount of water and dried. The dried sample was activated by nitrogen for one hour at 800 °C (heating rate 10 °C/min). After removing the sample from the oven, it was cooled to room temperature and washed with dilute HCl until it was defoamed by being heated together with water. It was then washed with distilled water before chlorine testing. Finally, the sample was ground and stored in plastic containers. All these processes resulted in 7 g of activated carbon being extracted from every 100 g of pumpkin seed shells.

### 2.3. Characterization of the Adsorbent

The Fourier Transform-Infrared Spectroscopy (FT-IR, Bruker Vertex 70v, Billerica, MA, USA) analysis was performed to demonstrate that the activated carbon obtained from the pumpkin seed shell was functionalized with KOH. Scanning Electron Microscopy (SEM, Zeiss EVO 10, Oberkochen, Germany) analysis was used to examine the morphological structure of the adsorbent in detail. The elemental analysis technique (CHNS-932, Leco, Corporation, St. Joseph, MI, USA) was applied to determine the adsorbent’s elemental composition. Further, the adsorbent’s net load was determined using Zeta analysis (Malvern/Zetasizer Nano ZSP, United Kingdom). Additionally, X-ray Photoelectron Spectroscopy (XPS, PHI 5000 VersaProbe, Chanhassen, MN, USA) was utilized to measure the elemental composition of the material’s surface of the material and the binding states of the elements. Brunauer–Emmett–Teller (BET, Nova 4200e Quantachrome Instruments, Boynton Beach, FL, USA) analysis was performed to determine the adsorbent’s surface area of the adsorbent.

### 2.4. Adsorption Studies

Batch system was preferred for CIP removal from aqueous systems. Adsorption studies were conducted according to the interaction time, adsorbent amount, pH and temperature parameters. Afterwards, practical applications continued under optimized conditions. UV-VIS spectrometer (UV-VIS 754, Shanghai Sunny Hengping Instrument Co., Ltd., Shanghai, China) was used to determine the amount of CIP before and after adsorption and was studied at a wavelength of 275 nm [29]. The adsorption (%) was calculated using the following equation:

(1) $$\mathrm{Adsorption}\left(\%\right)=\frac{\left(C_{o}-C_{e}\right)}{C_{o}}\times 100$$

In Equation (1), C_o_ is the initial concentration of CIP in solution (mg·L^−1^); C_e_ is the final CIP concentration in solution (mg·L^−1^).

The interaction time study was conducted first as part of the adsorption works. Measurements were taken over a period of 1–90 min, with each sample containing 1.5 mL and centrifuged at 3000 rpm. Then, the focus was on the determining the optimum amount of adsorbent (5–50 mg). The pH study utilized experimental applications in the range of 3.0–9.0. Finally, adsorption experiments were conducted at three different temperatures (15, 30, 45 °C) and under predetermined optimum conditions.

## 3. Results

### 3.1. Characterization

**Figure 1:**
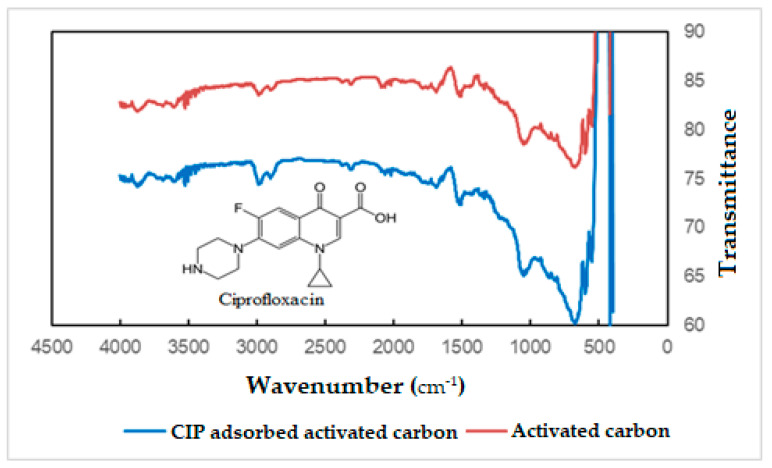
FT–IR spectra of activated carbons.

The effect of the CIP adsorption on the IR spectra is shown in Figure 1. The spectra of bare activated carbon and CIP adsorbed activated carbon were examined, and no significant difference was found. In both spectra, the band produced from the group –OH is apparent in the region 3450–3700 cm^−1^. The C–H bending vibration and C–O stretching peaks (2700–3000, 1250–1500, 500–700 cm^−1^) should be especially highlighted. The peak at 1690 cm^−1^ can occur from the C=C group. However, the intensity of the C–H bands in the spectrum of CIP adsorbed activated carbon was found to be greater. This may be due to CIP adsorbed onto the structure. Since, there, the CIP compound has numerous C–H bonds, it is expected that the C–H band intensity of the CIP adsorbed structure will be greater.

**Figure 2:**
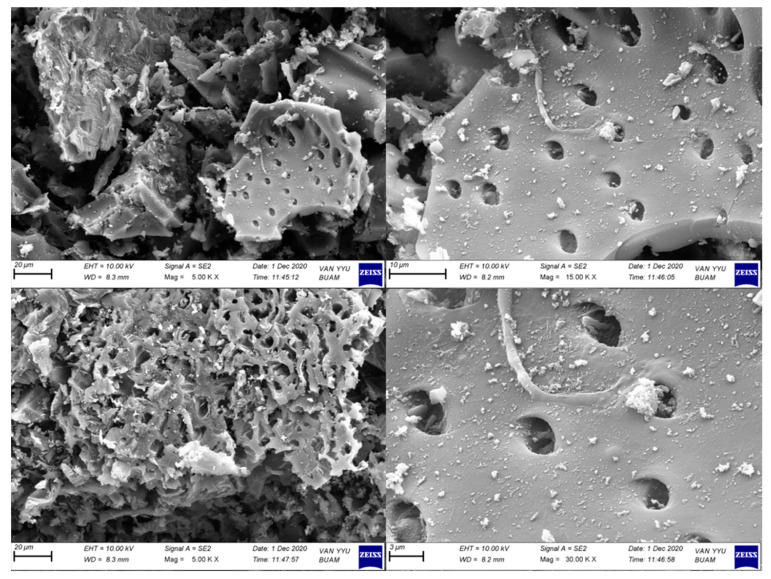
SEM images of modified activated carbon.

When the adsorbent’s SEM analysis is examined, it is particularly striking that it has a porous structure (Figure 2). The porous nature of construction is considered a critical advantage for adsorption [30,31,32,33,34], since the target molecule’s attachment to and its retention in the pore during interaction can be interpreted as a characteristic that facilitates adsorption. In addition, the fact that the adsorbed molecule is desorbed from the surface by various external factors complicates interactions within the pore. The average particle size was determined to be 50 µm.

Elemental analysis results of the activated carbon are given in Table 1. According to the data obtained, the sum of the elements carbon, hydrogen and nitrogen constitutes approximately 98.5% of the total mass. The high carbon content is gratifying in terms of the material obtained.

**Figure 3:**
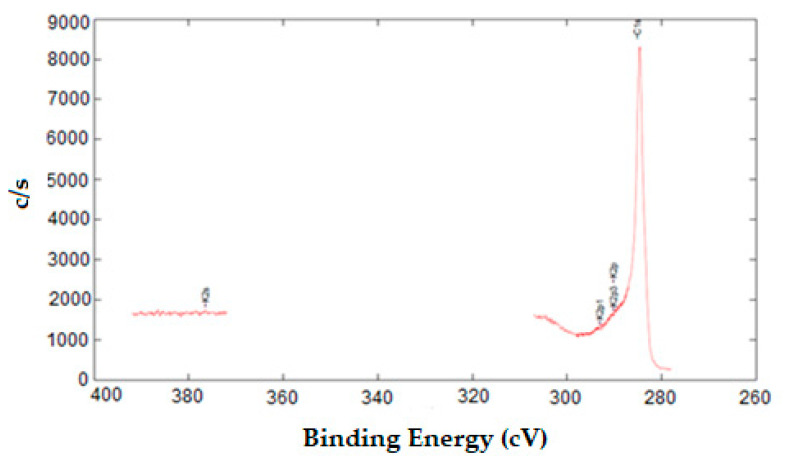
XPS spectrum of KOH modified activated carbon.

XPS was used to determine the chemical composition of the surface. Potassium and carbon atoms are identified in the spectrum which is interpreted as a result of the absorbent being treated with KOH treatment (Figure 3).

As a result of Zeta analysis, the potential of the adsorbent at pH 8.0 was calculated as 18.5 mV. In addition, the isoelectric point of the adsorbent was found to be 9.41. As a result, the adsorbent is uncharged at pH 9.41, positive at pH values less than 9.41 and negatively charged at pH values above 9.41. At the end of BET analysis, the adsorbent’s surface area was found to be 2729.7 m^2^·g^−1^. This number is extremely high, indicating that the adsorption ability of the adsorbent is very high. Previous studies have reported a surface area of 2000 mg·g^−1^ [35]. The surface area and maximum adsorption capacity of activated carbons obtained in earlier investigations as a result of KOH activation are presented in Table 2. As can be seen, the findings of this study are easily comparable with the existing literature and are quite successful.

### 3.2. Adsorption

**Figure 4:**
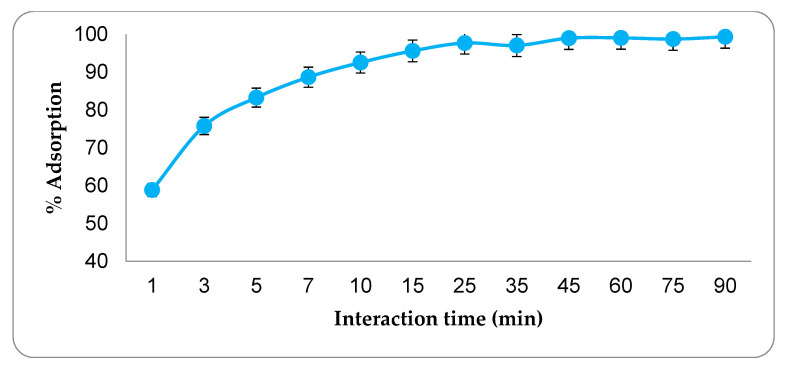
The effect of interaction time on the amount of adsorption. (CCIP: 40 mg·L^−1^, adsorbent amount: 37.5 mg, temperature: 300 °C).

**Figure 5:**
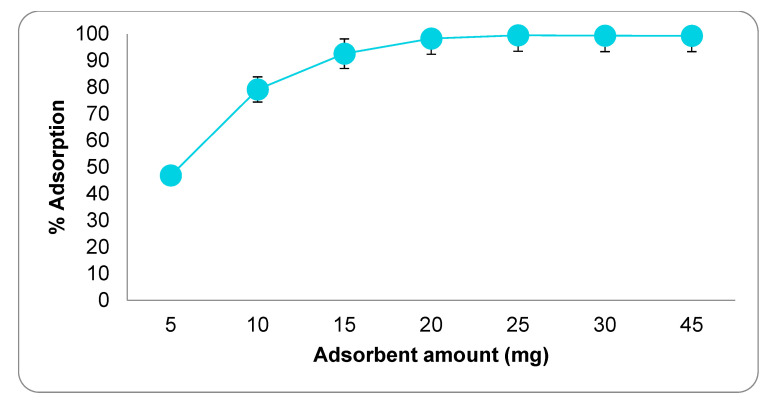
The effect of the amount of adsorbent on adsorption. (CCIP: 100 mg·L^−1^, interaction time: 45 min, temperature: 300 °C).

**Figure 6:**
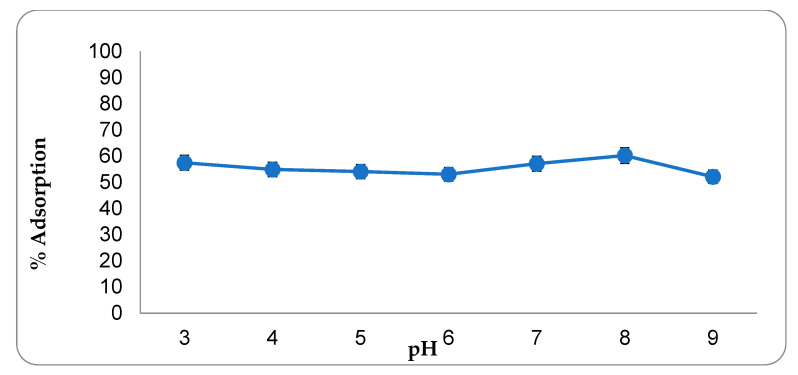
The effect of pH change on adsorption. (CCIP: 300 mg·L^−1^, interaction time: 45 min, adsorbent amount: 20.0 mg, temperature: 300 °C).

**Figure 7:**
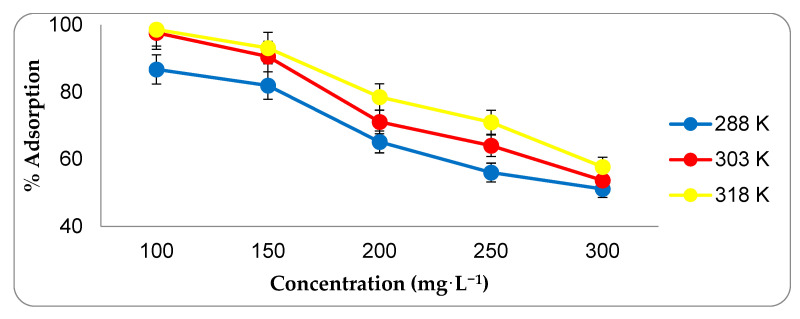
The effect of temperature change on adsorption. (Interaction time: 45 min, adsorbent amount: 20.0 mg, pH: 8.0).

In studies aimed at optimizing the adsorption conditions, the optimum interaction time was determined first. The results of the adsorption experiments conducted between 1 and 90 min are presented in Figure 4. As can be seen, the amount of CIP adsorbed increased significantly up to the 45th minute, but no significant increase in the amount of CIP adsorbed was found beyond this minute. The reason for this situation is that the adsorption regions of the adsorbent are completely saturated with CIP. The study conducted to determine the optimum adsorbent amount discovered that after 20 mg of adsorbent, the adsorption rate was nearly constant (Figure 5). Almost all of the CIP in the environment at this stage has been adsorbed by active carbon. As a result, 20 mg of activated carbon was determined as the optimal adsorbent amount. Adsorption studies carried out at various pH values revealed that the highest adsorption rate occurs at pH 8.0 point (Figure 6). Since the adsorbent’s isoelectric point is pH 9.41, it is positively charged for all pH values (pH 3–9) used in this investigation. The adsorption capacity between these points varies between 53–60 mg/g, indicating that the adsorption mechanism is predominantly non-ionic. This is because, despite being evaluated across a wide pH range, the obtained adsorption capacity values are not significantly different. Additionally, CIP adsorption at various concentrations was conducted at three different temperatures (Figure 7). As seen in the graph, the rate of CIP removal increased with increasing temperature in adsorption applications using the same concentration of solution. As a result, the interactions that are beneficial for adsorption are non-ionic interactions (hydrophobic). The functional structures of activated carbon, particularly the benzene ring and cyclopropane groups in the CIP molecule, establish an adsorption mechanism based on hydrophobic interaction.

### 3.3. Isotherm Studies

Linear adsorption isotherm models (Freundlich, Langmuir-1, Langmuir-2, Langmuir-3, Langmuir-4, Langmuir-5, Temkin and Dubinin–Radushkevich (D–R)) were used to explain the structure (surface properties, adsorption mechanism and capacity) of CIP adsorption on active carbon at 288, 303 and 313 K. The equations and parameters for the linear isotherm models used are shown in Table 3 [39,40,41,42].

Equilibrium data were applied to the five linear Langmuir isotherm model equations and the Linear Freundlich isotherm model. Correlation between the equilibrium data and the Langmuir-1 isotherm model was found to be high (R^2^) (Table 3). The Langmuir model is based on the assumption of homogeneous adsorption energies to the surface and the absence of adsorbate transition in the surface plane [40,43,44,45,46,47,48,49,50].

CIP’s adsorption data on active carbon were evaluated to fit five linearized expressions of the Langmuir isotherm model. Table 3 details these five distinct forms of linearized Langmuir equations and the values of Langmuir constants q_m_ (maximum adsorption capacity) and K_L_ (adsorption equilibrium constant). Among the correlation coefficient values of five different types of linearized Langmuir isotherm equations, Langmuir-1 was the most appropriate. The Langmuir-1 isotherm is typical of microporous adsorbents (activated carbons, zeolites) [51]. The maximum adsorption capacity was found to increase with increasing temperature, as seen by the decrease in the capacity values with decreasing temperature. This demonstrates the endothermic nature of the ongoing process once more [52]. Maximum adsorption capacity values determined using Langmuir-1 expression are higher than the amounts experimentally adsorbed and correspond to adsorption isotherm plateaus. On the contrary, it is clear that the Langmuir-1 isotherm more closely reflects the experimental data when the single-layer adsorption capacity and correlation coefficients are calculated using the other four Langmuir model linear expressions [40]. This result demonstrates that adsorption occurs in a single layer and is homogeneous [53,54].

Freundlich’s constant (K_F_), depending on the adsorption capacity, ranged from 265.58 mg·g^−1^ to 418.56 mg·g^−1^ with the temperature range studied. The value of K_F_ increased with increasing temperature, indicating that the adsorption interaction is endothermic. The n value is a constant that determines the type of process: If n = 1, adsorption is linear; if n < 1, adsorption is a chemical process; if n > 1, adsorption is a physical process. From Table 3, the 1/n value is 0.2152, 0.2079 and 0.1736 at 288, 303 and 318 K, respectively. Thus, the n value in this study was determined as 4.6472, 4.8094 and 5.7614 for all temperatures examined. The n > 1 case was the most common. It can be caused by any factor that causes the distribution of surface areas or by a decrease in the adsorbent–adsorbate interaction with increased surface density. Values in the range 1–10 indicate proper CIP adsorption and physical adsorption on active carbon [55].

According to the Temkin isotherm, the heat of adsorption decreases linearly for all molecules. This demonstrates the homogeneity of the binding energy [56]. While the typical binding energy range for the ion exchange mechanism is reported to be in the 8–16 kJ·mol^−1^ range, physical absorption processes are said to have adsorption energies less than −40 kJ·mol^−1^. The shallow b values (0.0190–0.0222 kJ·mol^−1^) obtained in this study indicate a weak ionic interaction between the sorbate and the existing sorbent, and the removal of CIP appears to involve physisorption [57].

The Langmuir and Freundlich isotherms are insufficient for clarifying the physical and chemical properties of adsorption. The D–R isotherm is more general than the Langmuir isotherm in that it does not assume a homogeneous surface or a constant sorption potential [58]. Average adsorption energy (E) provides information about chemical and physical adsorption. When the size of E is <8 kJ·mol^−1^, the adsorption process is called physical adsorption, and when E is between 8 kJ·mol^−1^ and 16 kJ·mol^−1^, the process is called chemical adsorption [59]. As seen in Table 3, the adsorption interaction is physical in nature due to the E value being <8 kJ·mol^−1^.

The thermodynamic properties of CIP adsorption on active carbon were determined using the ΔG°, ΔH° and ΔS° (entropy change) [41]. In order to find the Gibbs free energy of the adsorption process performed at a certain temperature, the equilibrium constant K_c_ is calculated using Equation (2).

K_c_ = C_a_/C_e_ (2)

K_c_: Equilibrium constant
C_a_: Concentration of substance retained by the adsorbent (mg·L^−1^)
C_e_: Concentration of residual substance in solution (mg·L^−1^)

ΔG° = −R·T·lnK_c_ (3)

(4) $${\mathrm{lnK}}_{c}=\frac{\Delta S^{{}^{\circ}}}{R}-\frac{\Delta H^{{}^{\circ}}}{\mathrm{RT}}$$

Here, R (8.314 J·mol ^−1^·K ^−1^) denotes the ideal gas constant, and T (K) is the temperature at Kelvin. ΔH° denotes the enthalpy change, and ΔS° denotes the entropy change in a particular process.

If K_c_, found with the help of Equation (2), is replaced in Equation (3), the Gibbs free energy of adsorption is determined.

Equation (4) calculates ∆H° from the slope of the line formed by plotting the lnKc value against the 1/T value, and ∆S° from the cut-off point. The values of ΔG°, ΔH° and ΔS° for CIP adsorption on activated carbon are given in Table 4.

As shown in Table 3, the values of ΔH° are positive, indicating an endothermic adsorption reaction [57,60,61]. Negative ΔG° values reflect the degree to which the adsorption process is spontaneous, whereas a more negative value indicates an energetically positive adsorption process. The decrease in ΔG° with increasing temperature indicated that adsorption at high temperature is more suitable. The value of G° was found to be negative for the adsorption of CIP on activated carbon at all temperatures, confirming the adsorbent’s applicability and the spontaneity of the adsorption process [57].

## 4. Conclusions

Within the scope of the study, CIP removal from aqueous systems was achieved at a high rate with activated carbon obtained from pumpkin seed shell. The fact that the adsorbent has a very large surface area has been one of the most outstanding aspects of the endeavor. The equilibrium adsorption data for the CIP aqueous solution indicate that the Langmuir model, which provides the best adsorption, is monolayered and homogeneous. However, the fact that the q_m_ value increases as temperature increases reveals that the adsorption process is endothermic. In the Freundlich model, the K_F_ value increases with increasing temperature, indicating that the adsorption interaction is endothermic. Values of the n value in the range of 1–10 show that it is suitable for CIP adsorption on activated carbon and represent physical adsorption on activated carbon. Since the E value is lower than 8 kJ·mol^−1^ in the Dubinin–Radushkevich (D–R) model, the adsorption process is physical. Since adsorption enthalpy values are less than 40 kJ·mol^−1^ in all cases studied, the fact that the sorption process is regulated by a physical rather than a chemical mechanism is confirmed [62]. It has been demonstrated that the adsorption process is endothermic, occurring physically and spontaneously. As a result, activated carbon has been demonstrated to be capable of being used as a low-cost and effective adsorbent for CIP removal.

## Figures and Tables

**Table 1:** Elemental analysis results of the adsorbent.

Element Name	Amount
Nitrogen	1.2331
Carbon	96.9629
Hydrogen	0.313
Totals	98.509

**Table 2:** Comparison of the specific surface area (BET) and maximum adsorption capacities (q_m_) for CIP on different activated carbon materials prepared via KOH activation.

Adsorbent	BET (m^2^·g^−1^)	q_m_ (mg·g^−1^)	References
*Albizia lebbeck* seed pods	1825	131.14	[21]
Smoked cigarette filters (CFAC-5)	2726	464.4	[36]
Long-root Eichhornia crassipes	-	145.00	[37]
Potato stems and leaves (APB)	38.75	23.36	[38]
Pumpkin seeds	2729.7	884.90	This study

**Table 3:** Isotherm models.

Isotherm	Linear Form	Temp. (K)	Constant Parameters			
Freundlich	${\mathrm{lnq}}_{e}={\mathrm{lnK}}_{F}+\frac{1}{n}{\mathrm{lnC}}_{e}$		**n**	**1/n**	**K_F_ (mg·g^−1^)**	**R^2^**
		288	4.6472	0.2152	265.58	0.8732
		303	4.8094	0.2079	311.35	0.8902
		318	5.7614	0.1736	418.46	0.9470
Langmuir-1	$\frac{1}{q_{e}}=\left(\frac{1}{K_{L}q_{m}}\right)\frac{1}{C_{e}}+\frac{1}{q_{m}}$		**K_L_ (L·mg^−1^)**	**q_m_ (mg·g^−1^)**	**R^2^**	
		288	0.0836	804.88	0.994	
		303	0.2781	819.61	0.9972	
		318	0.4176	884.90	0.9983	
Langmuir-2	$\frac{C_{e}}{q_{e}}=\frac{1}{q_{m}}C_{e}+\frac{1}{K_{L}q_{m}}$		**K_L_ (L·mg^−1^)**	**q_m_ (mg·g^−1^)**	**R^2^**	
		288	0.0951	792.56	0.9537	
		303	0.7119	774.08	0.9598	
		318	1.0181	832.22	0.9502	
Langmuir-3	$q_{e}=q_{m}-\left(\frac{1}{K_{L}}\right)\frac{q_{e}}{C_{e}}$		**K_L_ (L·mg^−1^)**	**q_m_ (mg·g^−1^)**	**R^2^**	
		288	0.1013	784.18	0.8899	
		303	0.6941	777.83	0.9119	
		318	0.9797	838.99	0.8881	
Langmuir-4	$\frac{q_{e}}{C_{e}}=K_{L}q_{m}-K_{L}q_{e}$		**K_L_ (L·mg^−1^)**	**q_m_ (mg·g^−1^)**	**R^2^**	
		288	0.0901	802.84	0.8899	
		303	0.6329	785.65	0.4683	
		318	0.8701	850.73	0.8881	
Langmuir-5	$\frac{1}{C_{e}}={\;K}_{L}q_{m}\frac{1}{q_{e}}-K_{L}$		**K_L_ (L·mg^−1^)**	**q_m_ (mg·g^−1^)**	**R^2^**	
		288	0.0894	804.280	0.9537	
		303	0.6790	778.930	0.9379	
		318	0.9588	839.610	0.9502	
Temkin	$q_{e}=B_{T}{\mathrm{lnK}}_{T}+B_{T}{\mathrm{lnC}}_{e}$ $B_{T}=\frac{\mathrm{RT}}{b}$		**b (kJ·mol^−1^)**	**K_T_ (L·mg^−1^)**	**R^2^**	
		288	0.0190	2.9322	0.9043	
		303	0.0192	4.5938	0.9264	
		318	0.2220	22.1366	0.9754	
Dubinin–Radushkevich	${\mathrm{lnq}}_{e}={\mathrm{lnq}}_{m}-{\beta \varepsilon }^{2}$ $\varepsilon =\mathrm{RTln}\left[1+\frac{1}{C_{e}}\right]$ $E_{a}=\left[\frac{1}{\sqrt{2\beta }}\right]$		**q_m_ (mg·g^−1^)**	**E (kJ·mol^−1^)**	**R^2^**	
		288	717.21	0.1622	0.9424	
		303	780.25	0.2427	0.943	
		318	836.73	0.6597	0.8905	

**Table 4:** Thermodynamic data for CIP adsorption.

C_o_	ΔH°, kJ·mol^−1^	ΔS°, J·mol^−1^	ΔG°, kJ·mol^−1^		
			288 K	303 K	313 K
100	39.39	150.87	−4.34	−5.72	−8.93
150	25.71	101.54	−3.63	−4.84	−6.70
200	18.77	70.14	−1.53	−2.27	−3.66
250	16.43	59.14	−0.61	−1.45	−2.39
300	9.62	33.85	−0.16	−0.58	−1.18

## Data Availability

All data are included in the article itself. There is no separate data sheet available.

## Sample Availability

Samples of the compounds are not available from the authors.

## References

[1] Darweesh T.M., Ahmed M.J. (2017). Adsorption of ciprofloxacin and norfloxacin from aqueous solution onto granular activated carbon in fixed bed column. Ecotoxicol. Environ. Saf..

[2] Ashfaq M., Khan K.N., Rasool S., Mustafa G., Saif-Ur-Rehman M., Nazar M.F., Sun Q., Yu C.-P. (2016). Occurrence and ecological risk assessment of fluoroquinolone antibiotics in hospital waste of Lahore, Pakistan. Environ. Toxicol. Pharmacol..

[3] Pouretedal H., Sadegh N. (2014). Effective removal of amoxicillin, cephalexin, tetracycline and penicillin G from aqueous solutions using activated carbon nanoparticles prepared from vine wood. J. Water Process Eng..

[4] Prutthiwanasan B., Phechkrajang C., Suntornsuk L. (2016). Fluorescent labelling of ciprofloxacin and norfloxacin and its application for residues analysis in surface water. Talanta.

[5] Van Doorslaer X., Dewulf J., Van Langenhove H., Demeestere K. (2014). Fluoroquinolone antibiotics: An emerging class of environmental micropollutants. Sci. Total Environ..

[6] Sun Y., Li H., Li G., Gao B., Yue Q., Li X. (2016). Characterization and ciprofloxacin adsorption properties of activated carbons prepared from biomass wastes by H_3_PO_4_ activation. Bioresour. Technol..

[7] Carabineiro S., Thavorn-Amornsri T., Pereira M., Serp P., Figueiredo J. (2012). Comparison between activated carbon, carbon xerogel and carbon nanotubes for the adsorption of the antibiotic ciprofloxacin. Catal. Today.

[8] Zhu L., Santiago-Schübel B., Xiao H., Hollert H., Kueppers S. (2016). Electrochemical oxidation of fluoroquinolone antibiotics: Mechanism, residual antibacterial activity and toxicity change. Water Res..

[9] Čvančarová M., Moeder M., Filipová A., Cajthaml T. (2015). Biotransformation of fluoroquinolone antibiotics by ligninolytic fungi–Metabolites, enzymes and residual antibacterial activity. Chemosphere.

[10] Sturini M., Speltini A., Maraschi F., Pretali L., Ferri E.N., Profumo A. (2015). Sunlight-induced degradation of fluoroquinolones in wastewater effluent: Photoproducts identification and toxicity. Chemosphere.

[11] Feng M., Wang X., Chen J., Qu R., Sui Y., Cizmas L., Wang Z., Sharma V.K. (2016). Degradation of fluoroquinolone antibiotics by ferrate (VI): Effects of water constituents and oxidized products. Water Res..

[12] Ebrahimpour B., Yamini Y., Moradi M. (2012). Application of ionic surfactant as a carrier and emulsifier agent for the microextraction of fluoroquinolones. J. Pharm. Biomed. Anal..

[13] Guo H., Gao N., Yang Y., Zhang Y. (2016). Kinetics and transformation pathways on oxidation of fluoroquinolones with thermally activated persulfate. Chem. Eng. J..

[14] Ferreira V.R., Amorim C.L., Cravo S.M., Tiritan M.E., Castro P.M., Afonso C.M. (2016). Fluoroquinolones biosorption onto microbial biomass: Activated sludge and aerobic granular sludge. Int. Biodeterior. Biodegrad..

[15] Tan F., Sun D., Gao J., Zhao Q., Wang X., Teng F., Quan X., Chen J. (2013). Preparation of molecularly imprinted polymer nanoparticles for selective removal of fluoroquinolone antibiotics in aqueous solution. J. Hazard. Mater..

[16] Depci T., Alkan S., Kul A., ÖNAL Y., Alacabey I., Dişli E. (2011). Characteristic properties of adsorbed catalase onto activated carbon based adiyaman lignite. Fresenius Environ. Bull..

[17] Maraschi F., Sturini M., Speltini A., Pretali L., Profumo A., Pastorello A., Kumar V., Ferretti M., Caratto V. (2014). TiO_2_-modified zeolites for fluoroquinolones removal from wastewaters and reuse after solar light regeneration. J. Environ. Chem. Eng..

[18] Sturini M., Speltini A., Maraschi F., Profumo A., Tarantino S., Gualtieri A.F., Zema M. (2016). Removal of fluoroquinolone contaminants from environmental waters on sepiolite and its photo-induced regeneration. Chemosphere.

[19] Liang Z., Zhaob Z., Sun T., Shi W., Cui F. (2016). Adsorption of quinolone antibiotics in spherical mesoporous silica: Effects of the retained template and its alkyl chain length. J. Hazard. Mater..

[20] Yu F., Li Y., Han S., Ma J. (2016). Adsorptive removal of antibiotics from aqueous solution using carbon materials. Chemosphere.

[21] Ahmed M.J., Theydan S.K. (2014). Fluoroquinolones antibiotics adsorption onto microporous activated carbon from lignocellulosic biomass by microwave pyrolysis. J. Taiwan Inst. Chem. Eng..

[22] Almaz Kemal K.S., Michael W.H. (2019). Adsorption of Cu (II) and Cd (II) onto Activated Carbon Prepared from Pumpkin Seed Shell. J. Environ. Sci. Pollut. Res..

[23] Demiral İ., Şamdan C.A. (2016). Preparation and characterisation of activated carbon from pumpkin seed shell using H_3_PO_4_. Anadolu Univ. J. Sci. Technol. A-Appl. Sci. Eng..

[24] Kuśmierek K., Świątkowski A., Dąbek L. (2017). Removal of 2, 4, 6-trichlorophenol from aqueous solutions using agricultural waste as low-cost adsorbents. Environ. Prot. Eng..

[25] Demiral İ., Bektaş T., Şamdan C. (2019). Utilization of activated carbon prepared from pumpkin seed shell for the removal of dyestuff from aqueous solutions and wastewater by microwave radiation. Int. J. Sci. Technol. Res..

[26] Demiral I., Aydın Şamdan C., Demiral H. (2016). Production and characterization of activated carbons from pumpkin seed shell by chemical activation with ZnCl_2_. Desalin. Water Treat..

[27] Huang G.G., Liu Y.-F., Wu X.X., Cai J.J. (2019). Activated carbons prepared by the KOH activation of a hydrochar from garlic peel and their CO_2_ adsorption performance. New Carbon Mater..

[28] Le Van K., Thu T.L.T., Thu H.N.T., Van Hoang H. (2019). Activated Carbon by KOH and NaOH Activation: Preparation and Electrochemical Performance in K_2_SO_4_ and Na_2_SO_4_ Electrolytes. Russ. J. Electrochem..

[29] Cazedey E.C.L., Salgado H.R.N. (2012). Spectrophotometric determination of ciprofloxacin hydrochloride in ophthalmic solution. Adv. Anal. Chem..

[30] Erol K., Uzun L. (2017). Two-step polymerization approach for synthesis of macroporous surface ion-imprinted cryogels. J. Macromol. Sci. Part A.

[31] Erol K., Bolat M., Tatar D., Nigiz C., Köse D.A. (2020). Synthesis, characterization and antibacterial application of silver nanoparticle embedded composite cryogels. J. Mol. Struct..

[32] Erol K., Tatar D., Veyisoğlu A., Tokatlı A. (2020). Antimicrobial magnetic poly (GMA) microparticles: Synthesis, characterization and lysozyme immobilization. J. Polym. Eng..

[33] Erol K. (2017). Synthesis, Characterization and Chromatographic Applications of Antimicrobial Cryogels. Hacet. J. Biol. Chem..

[34] Ece M.Ş. (2021). Synthesis and characterization of activated carbon supported magnetic nanoparticles (Fe_3_O_4_/AC@SiO_2_@Sulfanilamide) and its application in removal of toluene and benzene. Colloids Surf. A Physicochem. Eng. Asp..

[35] Yang K., Peng J., Srinivasakannan C., Zhang L., Xia H., Duan X. (2010). Preparation of high surface area activated carbon from coconut shells using microwave heating. Bioresour. Technol..

[36] Zhang X., Xu J., Lv Z., Wang Q., Ge H., Wang X., Hong B. (2020). Preparation and utilization of cigarette filters based activated carbon for removal CIP and SDS from aqueous solutions. Chem. Phys. Lett..

[37] Liu L., Chen X., Wang Z., Lin S. (2019). Removal of aqueous fluoroquinolones with multi-functional activated carbon (MFAC) derived from recycled long-root Eichhornia crassipes: Batch and column studies. Environ. Sci. Pollut. Res..

[38] Li R., Wang Z., Guo J., Li Y., Zhang H., Zhu J., Xie X. (2018). Enhanced adsorption of ciprofloxacin by KOH modified biochar derived from potato stems and leaves. Water Sci. Technol..

[39] Acet Ö., Baran T., Erdönmez D., Aksoy N.H., Alacabey İ., Menteş A., Odabaşi M. (2018). O-carboxymethyl chitosan Schiff base complexes as affinity ligands for immobilized metal-ion affinity chromatography of lysozyme. J. Chromatogr. A.

[40] Hamdaoui O., Naffrechoux E. (2007). Modeling of adsorption isotherms of phenol and chlorophenols onto granular activated carbon: Part I. Two-parameter models and equations allowing determination of thermodynamic parameters. J. Hazard. Mater..

[41] Banerjee P., Sau S., Das P., Mukhopadhayay A. (2015). Optimization and modelling of synthetic azo dye wastewater treatment using graphene oxide nanoplatelets: Characterization toxicity evaluation and optimization using artificial neural network. Ecotoxicol. Environ. Saf..

[42] Wakkel M., Khiari B., Zagrouba F. (2019). Basic red 2 and methyl violet adsorption by date pits: Adsorbent characterization, optimization by RSM and CCD, equilibrium and kinetic studies. Environ. Sci. Pollut. Res..

[43] Erol K. (2017). The adsorption of calmoduline via nicotinamide immobilized poly (HEMA-GMA) cryogels. J. Turk. Chem. Soc. Sect. A Chem..

[44] Erol K. (2017). Polychelated cryogels: Hemoglobin adsorption from human blood. Artif. Cells Nanomed. Biotechnol..

[45] Erol K., Yıldız E., Alacabey İ., Karabörk M., Uzun L. (2019). Magnetic diatomite for pesticide removal from aqueous solution via hydrophobic interactions. Environ. Sci. Pollut. Res..

[46] Erol K. (2016). DNA adsorption via Co (II) immobilized cryogels. J. Macromol. Sci. Part A.

[47] Erol B., Erol K., Gökmeşe E. (2019). The effect of the chelator characteristics on insulin adsorption in immobilized metal affinity chromatography. Process Biochem..

[48] Kireç O., Alacabey İ., Erol K., Alkan H. (2021). Removal of 17β-estradiol from aqueous systems with hydrophobic microspheres. J. Polym. Eng..

[49] Alacabey İ., Acet Ö., Önal B., Dikici E., Karakoç V., Gürbüz F., Alkan H., Odabaşı M. (2021). Pumice particle interface: A case study for immunoglobulin G purification. Polym. Bull..

[50] Satır Tosun İ., Erol K. (2021). Calcined Eggshell for Removal of Victoria Blue R Dye from Wastewater Medium by Adsorption. J. Turk. Chem. Soc. Sect. A Chem..

[51] Czepirski L., Balys M.R., Komorowska-Czepirska E. (2000). Some generalization of Langmuir adsorption isotherm. Internet J. Chem..

[52] Mittal A., Kurup L., Mittal J. (2007). Freundlich and Langmuir adsorption isotherms and kinetics for the removal of Tartrazine from aqueous solutions using hen feathers. J. Hazard. Mater..

[53] Alacabey İ., Kul A.R., Şakir E., Alkan H. (2020). Van Gölü Doğal Sediment ve Modifiye Sediment Üzerine Krom (III) Adsorpsiyonu (İzoterm ve Termodinamik Analiz Çalışması). Dicle Üniversitesi Mühendislik Fakültesi Mühendislik Dergisi.

[54] Ece M.S.A., Kutluay S., Şahin O.M., Horoz S. (2020). Development of novel Fe_3_O_4_/AC@SiO_2_@1, 4-DAAQ magnetic nanoparticles with outstanding VOC removal capacity: Characterization, optimization, reusability, kinetics, and equilibrium studies. Ind. Eng. Chem. Res..

[55] Shin H.S., Kim J.H. (2016). Isotherm, kinetic and thermodynamic characteristics of adsorption of paclitaxel onto Diaion HP-20. Process Biochem..

[56] ALOthman Z.A., Naushad M., Ali R. (2013). Kinetic, equilibrium isotherm and thermodynamic studies of Cr (VI) adsorption onto low-cost adsorbent developed from peanut shell activated with phosphoric acid. Environ. Sci. Pollut. Res..

[57] Kiran B., Kaushik A. (2008). Chromium binding capacity of Lyngbya putealis exopolysaccharides. Biochem. Eng. J..

[58] Caliskan N., Kul A.R., Alkan S., Sogut E.G., Alacabey I. (2011). Adsorption of Zinc (II) on diatomite and manganese-oxide-modified diatomite: A kinetic and equilibrium study. J. Hazard. Mater..

[59] Hu Q., Zhang Z. (2019). Application of Dubinin–Radushkevich isotherm model at the solid/solution interface: A theoretical analysis. J. Mol. Liq..

[60] Ngah W.W., Fatinathan S. (2008). Adsorption of Cu (II) ions in aqueous solution using chitosan beads, chitosan—GLA beads and chitosan—Alginate beads. Chem. Eng. J..

[61] Hu X.J., Wang J.S., Liu Y.G., Li X., Zeng G.M., Bao Z.L., Zeng X.X., Chen A.W., Long F. (2011). Adsorption of chromium (VI) by ethylenediamine-modified cross-linked magnetic chitosan resin: Isotherms, kinetics and thermodynamics. J. Hazard. Mater..

[62] Sogut E.G., Caliskan N. (2017). Removal of lead, copper and cadmium ions from aqueous solution using raw and thermally modified diatomite. Desalin. Water Treat..

